# Degree of resistance of *Solanum torvum* cultivars to *Mi-1.2*-virulent and avirulent isolates of *Meloidogyne incognita*, *Meloidogyne javanica*, and *Meloidogyne luci*

**DOI:** 10.21307/jofnem-2021-068

**Published:** 2021-07-30

**Authors:** Seren Sargın, Zübeyir Devran

**Affiliations:** 1 Department of Plant Protection, Faculty of Agriculture, Akdeniz University, Antalya, Turkey

**Keywords:** Eggplant, Resistance, Root-knot nematode, *Solanum torvum*

## Abstract

Root-knot nematodes (RKN) cause yield losses in eggplant-growing areas. There are no known varieties of eggplant (*Solanum melongena* L.) that are resistant to RKNs. However, the wild relative of eggplant, *S. torvum* (Sw.), provides resistance to some RKN species and is used as a rootstock for cultivated eggplants. Therefore, determination of the reproductive capacity of nematodes on eggplant rootstocks developed from *S. torvum* is required for effective management of RKNs that are widely present in vegetable growing areas. In the present study, the degree of reproduction of *Mi-1.2*-virulent and avirulent isolates of *M*. *incognita*, *M*. *javanica*, and *M*. *luci* on eggplant rootstocks, Hawk and Boğaç, was evaluated in a plant growth chamber. Hawk and Boğaç were resistant (< 10 egg masses per whole root system) to all avirulent and virulent isolates of *M*. *incognita*, *M*. *javanica*, and *M*. *luci.* This study is the first report on the resistance of *S. torvum* to virulent isolates of *M. luci*. Results indicate that *S. torvum* offers broad-spectrum resistance against RKNs.

Eggplant (*Solanum melongena* L.) is a member of the Solanaceae family and has wide genetic variation in shape, colour and size (Daunay et al., 2001). In 2019, eggplant was grown in 1.8 M hectares, with a total production of 55 M tons worldwide. Turkey is the world’s fourth largest eggplant producer after China, India, and Egypt, with an annual production of 8.2 M tons (FAO, 2021). Root-knot nematodes (RKNs) are one of the most important pathogens affecting eggplant. The use of plants resistant to RKNs is the most effective and environment-friendly tactic for management of this pathogen (Seid et al., 2015). Although there are no resistant cultivars of *S. melongena*, some wild eggplant species (*S. torvum*) have been found to be resistant to some RKNs (Ali et al., 1992; Mattos et al., 2011; Rahman et al., 2002). However, resistant hybrids of these wild species with cultivars have not been developed because of cross-incompatibility between the phylogenetically distant *S. torvum* and cultivated *S. melongena* (Daunay et al., 2001; McCammon and Honma, 1983). Grafting with resistant rootstocks is an additional technique that can alleviate the problem of soil borne disease. *Solanum torvum* has been employed as a rootstock to control RKNs in eggplant cultivation (Uehara et al., 2016, 2017), but it has variable responses to the major RKNs. *Meloidogyne arenaria* (A2-J) (Chitwood, 1949; Neal, 1889) produced large numbers of egg masses on *S. torvum* root stock cv. Tonashimu, but *M. arenaria* (A2-O) and *M. incognita* produced only a few egg masses (Uehara et al., 2017). A study conducted by Garcia-Mendivil et al. (2019) showed that *S. torvum* rootstock cv. Brutus, Espina, Salutamu and Torpedo were resistant to *M. incognita* isolates and varied from highly resistant to susceptible to *Mi-1.2* (a)virulent isolates of *M. javanica* (Chitwood, 1949; Treub, 1885) from Spain. In other studies, *S. torvum* cv. Hawk was found to be resistant to *M. incognita* (Chitwood, 1949; Kofoid and White, 1919) (both *Mi-1.2-*virulent and avirulent isolates), *M. javanica, M. arenaria*, and *M. luci* (Carneiro et al., 2014) isolates, but susceptible to isolate of *M. hapla* (Chitwood, 1949) from Turkey (Öçal and Devran, 2019; Öçal et al., 2018). Recently, Murata and Uesugi (2020) reported that *S. torvum* cvs. Torvum vigor, Tonashimu and Torero were susceptible to three *M. hapla* populations collected from geographically distant areas in Japan.

In tomato, resistant cultivars are widely used to control RKNs. The *Mi-1* gene in tomato confers resistance to *M. incognita*, *M. javanica*, and *M. arenaria* (Milligan et al., 1998; Roberts and Thomason, 1986; Williamson and Hussey, 1996), as well as *M. luci* (Aydınlı and Mennan, 2019). However, *Mi-*resistance-breaking *M. incognita* and *M. javanica* populations were reported in United States of America, Spain, Greece, Turkey, and Israel (Devran and Söğüt, 2010; Iberkleid et al., 2014; Kaloshian et al., 1996; Ornat et al., 2001; Tzortzakakis et al., 2005). In addition, a virulent population of *M. luci* has been reported from Turkey (Aydınlı and Mennan, 2019). *Meloidogyne luci* poses a phytosanitary risk as the species was included on the EPPO Alert List of harmful organisms in 2017 (EPPO, 2017). In previous studies, *S. torvum* cultivars Tonashimu, Torero, Torvum vigor, Hawk, Brutus, Espina, Salutamu, and Torpedo were found to be resistant to *Mi-1.2-*virulent isolates of *M. incognita* from Japan and Turkey and *Mi-1.2*-virulent *M. javanica* from Spain (Garcia-Mendivil et al., 2019). Thus, knowledge of the response of *S. torvum* rootstock cultivars to various RKN populations is important for control of both *Mi-1.2-*avirulent and virulent populations of root-knot nematodes, which are likely to be present in mixed populations in vegetable growing areas of Turkey. In Turkey, two *S. torvum* rootstocks, Hawk and Boğaçare used to control RKNs in eggplant growing areas. *Solanum torvum* cv. Hawk has been described as resistant to some RKN isolates from Turkey (Öçal et al., 2018). As far as the authors are aware, there is no information about the response of commercial *S. torvum* cultivars to *Mi-1.2* virulent isolates of *M. javanica* and *M. luci* from Turkey or the response of *S. torvum* cv. Boğaçto root-knot nematodes isolates. Here, we report the degree of resistance of *S. torvum* cultivars Hawk and Boğaç to avirulent and virulent isolates of *M. incognita*, *M. javanica,* and *M. luci* from Turkey.

## Materials and methods

### Plant material

The two *S. torvum* rootstocks Hawk and Boğaç and a commercial eggplant cultivar Faselis F_1_ were used in this study (Table 1). All seeds were purchased from companies. Seeds were sown in vials containing vermiculite, perlite, and peat (v:v:v, 1:1:1) and incubated in controlled environment at 25 ± 5°C. Seedlings with two true leaves were transplanted singly to 250 ml pots containing sterilized sandy soil (75% sand, 15% silt, and 10% clay) after germination. The plants were watered when needed.

**Table 1.** *Solanum melongena* (eggplant) and *Solanum torvum* rootstock cultivars used in this study.

Plant	Cultivar	Company
*Solanum torvum*	Hawk	Vilmorin Anadolu Tohumculuk
*Solanum torvum*	Bog˘aç	Yüksel Tohum Tarım San. ve Tic. A.S¸.
*Solanum melongena*	Faselis F_1_	Titiz Agrogrup A.S¸.

### Root-knot nematode culture

Four *M*. *incognita*, four *M*. *javanica*, and two *M*. *luci* isolates were used in the experiment (Table 2). The virulence and reproductive ability of *M*. *incognita*, *M*. *javanica*, and *M*. *luci* isolated from different hosts were determined on tomato cv. Seval F1 (Multi Seed, Turkey) carrying the *Mi-1.2* gene. Avirulent isolates of *M*. *incognita*, *M*. *javanica*, and *M*. *luci* were multiplied on susceptible tomato cv. Tueza F1 (Multi Seed, Turkey) and Falcon F1 (May Seed, Turkey) in previous studies (Aydınlı and Mennan, 2019; Devran and Söğüt, 2010). Each isolate was raised from single egg mass to obtain pure cultures. The plants were removed 60 days after root-knot nematode inoculation, and their roots were washed under water. Egg masses were picked from plant roots using a needle and incubated in a modified Baermann funnel technique (Hooper, 1986) at 25°C room temperature for 1 day. Then, the J2s hatched from the egg masses were collected and counted under a light microscope according to the method described in previous studies (Özalp and Devran, 2018; Öçal et al., 2018).

**Table 2.** Geographic origin and virulence status of root-knot nematode isolates from Turkey.

*Meloidogyne* spp.	Isolate	Original host	Geographic origin	(a)Virulence	Reference
*M*. *incognita*	G4	Eggplant	Gazipas¸a, Antalya	Avirulent	Devran and Sög˘üt (2009, 2010)
*M*. *incognita*	K5	Pepper	Kumluca, Antalya	Avirulent	Devran and Sög˘üt (2009, 2010)
*M*. *incognita*	V6	Tomato	Kepez, Antalya	*Mi-1.2* virulent	Laboratory Culture
*M*. *incognita*	V20	Tomato	Kepez, Antalya	*Mi-1.2* virulent	Mıstanog˘lu et al. (2020)
*M*. *javanica*	K21	Cucumber	Finike, Antalya	Avirulent	Devran and Sög˘üt (2009, 2010)
*M*. *javanica*	F4-1	Tomato	Fethiye, Mug˘la	Avirulent	Laboratory Culture
*M*. *javanica*	V26	Tomato	Kumluca, Antalya	*Mi-1.2* virulent	Mıstanog˘lu et al. (2020)
*M*. *javanica*	V28	Tomato	Aksu, Antalya	*Mi-1.2* virulent	Mıstanog˘lu et al. (2020)
*M*. *luci*	TK4	Nightshade	Tekkeköy, Samsun	Avirulent	Aydınlı and Mennan (2019)
*M*. *luci*	OR2	Tomato	Ordu	*Mi-1.2* virulent	Aydınlı and Mennan (2019)

### Nematode inoculation

Eggplant seedlings with three true leaves were inoculated with 1,000 one-day-old J2s. The J2s were dropped using a Pasteur pipette into a 2-cm deep hole around the stem base according to the method described in previous studies (Öçal and Devran, 2019; Öçal et al., 2018). Each nematode species-rootstock combination was replicated five times in a completely randomized design and the experiment was repeated twice under same conditions. The pots were maintained in a growth chamber at 24 ± 1°C with a 16:8 hr (light:dark) photoperiod and 65% relative humidity. Sixty days after inoculation, the plants were removed, and their roots were washed under water. Each root system was dipped in a 0.15 g/L phloxine B solution for 10 min to identify egg masses, according to Öçal et al. (2018).

### Data collection

The number of egg masses on each plant root was counted under a stereomicroscope and assessed on a 0–5 scale, according to Hartman and Sasser (1985), as follows: 0 = no egg mass (resistant), 1 = 1–2 egg masses (resistant), 2 = 3–10 egg masses (resistant), 3 = 11–30 egg masses (susceptible), 4 = 31–100 egg masses (susceptible), and 5 = more than 100 egg massesper root system (susceptible).

### Statistical analyses

Data on number of egg masses were analyzed using the SAS statistical software (SAS Institute, Cary, NC). Data were log transformed [log10(*x* + 1)] to homogenize the variances, and then subjected to analysis of variance (ANOVA). Separation of treatment means was performed using Tukey’s HSD tests (*p* < 0.05).

## Results

*Solanum torvum* rootstock cultivars Hawk and Boğaç, and a commercial eggplant cultivar Faselis F_1_ were tested with ten *Mi-1.2* virulent and avirulent isolates of *M. incognita*, *M. javanica*, and *M. luci*. *Meloidogyne incognita* V6 and *M. luci* TK4 isolates did not produce any egg masses on roots of Boğaç. However, the other RKN isolates produced a few egg masses (< 2) on both *S. torvum* rootstocks Hawk and Boğaç. All RKN isolates also produced egg masses (> 38) on roots of Faselis F_1_, with the highest number (> 280) produced by *M. javanica* isolate K5, and the least (< 39) by *M. luci* isolate TK4 (Table 3). Differences among the number of egg masses produced by RKN isolates on roots of seedlings were not statistically significant for *S. torvum* rootstocks Hawk and Boğaç, but the numbers on these rootstocks were significantly different from those on the eggplant cv. Faselis F_1_ (Table 3). Both Hawk and Boğaç were classed as resistant to all RKN isolates according to the 0–5 egg mass index, with scores less than 1 for all isolates, except the *M. incognita* G4 isolate. In contrast, eggplant cv. Faselis F_1_ was found to be susceptible, as expected, with scores for all RKN isolates greater than 3. Our data indicate that *S. torvum* rootstocks Hawk and Boğaç, are non-hosts for all RKN isolates tested. Also, for the first time, our data have shown that the two *S. torvum* rootstock cultivars were resistant to a *Mi-1.2-*virulent isolate of *M. luci*.

**Table 3.** Number of egg masses and egg mass index of *Meloidogyne incognita*, *Meloidogyne javanica*, and *Meloidogyne luci* isolates on the *Solanum torvum* rootstock cvs. Hawk and Boğaç and the eggplant cv. Faselis F_1_.

Nematode species	Isolate	Hawk EM EMI^1^		Boğaç EM EMI^1^		Faselis F_1_ EM EMI^1^	
*M. incognita*	G4	1.4B	0.9B	1.9B	1.2B	266.9A	5A
	K5	0.9B	0.7B	1.4B	0.9B	281.3A	5A
	V6	0.2B	0.2B	0B	0B	181.6A	5A
	V20	0.1B	0.1B	0.4B	0.3B	52.7A	3.8A
*M. javanica*	K21	0.9B	0.7B	0.7B	0.4B	264A	5A
	F4-1	0.4B	0.3B	0.4B	0.3B	135.4A	4.8A
	V26	0.5B	0.4B	0.5B	0.3B	135.6A	4.8A
	V28	0.8B	0.5B	0.7B	0.6B	185.4A	4.9A
*M. luci*	TK4	0.1B	0.1B	0B	0B	38.3A	3.7A
	OR2	0.3B	0.3B	0.1B	0.1B	66.1A	4.1A

**Notes:** EM: egg masses, EMI: egg mass index. ^1^0–5 Scale (Hartman and Sasser, 1985), 0 = no egg masses, 1 = 1–2 egg masses, 2 = 3–10 egg masses, 3 = 11–30 egg masses, 4 = 31–100 egg masses, 5 = more than 100 egg masses. Resistant: Egg mass index (EMI) ≤ 2; Susceptible: Egg mass index (EMI) > 2. Values within a line followed by the same upper case letter are not significantly different (*p* = 0.05) according to Tukey’s multiple range test. The data represent means of five replicates. Each nematode species-rootstock combination was replicated five times in a completely randomized design and the experiment was repeated twice. Sixty days after inoculation, the plants were removed and the egg masses were counted from the whole root system.

## Discussion

Eggplant is one of the most important vegetable crops for human nutrition. However, many pathogens and pests negatively affect vegetable production. Root-knot nematodes (RKNs) are one of the most important pathogens causing yield losses in vegetable growing areas. They inhibit the plant’s water and nutrient intake from soil and cause galls on its roots. Different management methods are used to control RKNs and use of resistant plants is one of the most important (Boerma and Hussey, 1992). Previous studies showed that *S. melongena* cultivars were susceptible to *M. incognita*, *M. javanica*, and *M. arenaria* (Daunay and Dalmasso, 1985; Devi and Sumita, 2015; Nayak and Pandey, 2015; Öçal and Devran, 2019; Ullah et al., 2011). The present study has confirmed the susceptibility of the commercial *S. melongena* cultivar Faselis F_1_ to all RKN isolates. However, results from this study show that *S. torvum* provides broad resistance to RKN isolates. We found resistance in the two *S. torvum* rootstock cultivars Hawk and Boğaç to all *Mi-1.2* virulent and avirulent isolates of *M*. *incognita*, *M*. *javanica*, and *M*. *luci* tested. This is in agreement with earlier studies that described wild *S. torvum* as resistant to *M. incognita*, *M. javanica*, *M. arenaria*, and *M. luci* (Dhivya et al., 2014; Öçal and Devran, 2019; Öçal et al., 2018; Tzortzakakis et al., 2006; Uehara et al., 2017) but susceptible to *M. hapla* (Murata and Uesugi, 2020; Öçal et al., 2018). Other studies also reported that *S. torvum* cultivars were resistant to *Mi-1.2* virulent *M. incognita* isolates from Japan (Uehara et al., 2016), a *Mi-1.2* virulent *M. incognita* isolate from Turkey (Öçalal et al., 2018) and *Mi-1.2* virulent *M. javanica* isolates from Spain (Garcia-Mendivil et al., 2019); however, although Öçal et al. (2018) reported resistance in *S. torvum* to an avirulent isolate of *M. luci* the present study provides the first evidence of resistance in *S. torvum* rootstocks to a *Mi-1.2* virulent isolates of *M. luci*. *Meloidogyne luci* has been reported to occur in Brazil, Chile, Iran (Carneiro et al., 2014), Guatemala (Janssen et al., 2016), Portugal (Maleita et al., 2017), and Turkey (Aydınlı and Mennan, 2019), and so sources of resistance to virulent isolates of this RKN are of importance to growers in these countries.

Applications of chemical control against plant parasitic nematodes are expensive and have been banned due to their adverse effects on the environment and harmful residual effects on human health. Therefore, resistant varieties and grafted seedlings are preferred due to their lower cost, their effectiveness, and their long-term and environmentally friendly protection compared to chemicals (Barrett et al., 2012; Cook and Starr, 2006; Lopes et al., 2019; Williamson and Kumar, 2006). Also, *Mi-1.2* virulent RKNs are becoming widespread in vegetable-growing areas. Therefore, new sources of resistance are essential for planting in fields infested with RKNs. The results of this study show that *S. torvum* is a non-host for *M. incognita*, *M. javanica*, and *M. luci* isolates from Turkey and is, therefore, a valuable tool for controlling *M. incognita*, *M. javanica*, and *M. luci* populations irrespective of their (a)virulence status to the *Mi-1.2* gene. Further studies are needed to test other *S. torvum* rootstock varieties to determine how their yields compare with those of susceptible commercial cultivars.
